# Case report: Rituximab combined with plasma exchange treatment for systemic lupus erythematosus complicated with thrombotic microangiopathy and non-cirrhotic portal hypertension

**DOI:** 10.3389/fimmu.2024.1475303

**Published:** 2025-01-03

**Authors:** Jinmei Huang, Wei Fan, Xuyan Chen, Shufan Wu, Zhigao Dong, Yi Zhang, Yiwan Lin, Pingping Xiao

**Affiliations:** Department of Rheumatology and Immunology, the Second Affiliated Hospital of Xiamen Medical College, Xiamen, China

**Keywords:** systemic lupus erythematosus, thrombotic microangiopathy, non-cirrhotic portal hypertension, rituximab, case report

## Abstract

**Introduction:**

Systemic lupus erythematosus (SLE) complicated by thrombotic microangiopathy (TMA) and non-cirrhotic portal hypertension (NCPH) is rare. We present a case of a female patient with SLE who developed TMA and NCPH and responded positively to rituximab and plasma exchange treatment.

**Case description:**

A 53-year-old woman was admitted with 6 h of confusion. Upon admission, she was diagnosed with SLE complicated by lupus encephalopathy, blood system impairment, cardiomyopathy, and nephritis. Initial treatment with high-dose methylprednisolone, immunoglobulin shock therapy, and tacrolimus (1 mg, twice daily) improved her symptoms and laboratory indicators. However, after a pulmonary infection and infection with the 2019 novel coronavirus, the patient’s condition deteriorated further. She experienced confusion and a delayed response. Hemoglobin levels and platelet counts decreased, lactate dehydrogenase and creatinine levels increased, and the percentage of peripheral schistocytes was approximately 6.5%. Abdominal ultrasonography revealed a substantial amount of ascites, diffuse liver lesions, splenomegaly, and splenic varices. Enhanced computed tomography revealed diffuse liver disease along the portal veins, intrahepatic lymphatic dilatation, esophageal and gastric varices, a splenorenal vein shunt, and splenomegaly. The patient was negative for hepatitis virus, autoimmune liver disease antibodies, ceruloplasmin, and tumor markers. Therefore, SLE complicated by TMA and NCPH was considered. She was treated with high-dose methylprednisolone (500 mg) for 3 days and immunoglobulin (0.4 g/kg/day) for 5 days, followed by rituximab (500 mg) for suppressive immunotherapy combined with plasma exchange (seven times), low-molecular-weight heparin (5,000 U every 12 h) for anticoagulation, and a diuretic. The patient’s symptoms and laboratory indicators improved.

**Conclusion:**

This case suggests that a combination of rituximab, plasma exchange, anticoagulation, and diuretics may be an effective treatment for patients with SLE complicated by TMA and NCPH.

## 1 Introduction

Systemic lupus erythematosus (SLE) is an autoimmune disease characterized by multi-organ and multi-system involvement (1). SLE complicated with thrombotic microangiopathy (TMA) and non-cirrhotic portal hypertension (NCPH) is rare in clinical practice. TMA is a kind of clinicopathologic syndrome with microangiopathic hemolytic anemia, thrombocytopenia, and organ damage mainly based on terminal arteriole and capillary endothelial injury and thrombosis (2). The pathological manifestations include extensive microvascular endothelial cell swelling, microthrombus formation, and capillary cavity stenosis. NCPH is a rare vascular liver disease with clinical manifestations of portal hypertension without cirrhosis or severe fibrosis. It is reported that NCPH is related to autoimmune diseases; however, only a few cases have been reported in SLE. Here, we report a case of a female patient with SLE complicated by TMA and NCPH.

## 2 Case description

A 53-year-old woman presented with confusion for 6 h. Physical examination revealed a blood pressure of 183/110 mmHg, oxygen saturation of 96%, body temperature of 36.2°C, heart rate of 92 beats/min, and respiratory rate of 16 breaths/min. She also presented with drowsiness, a pale face, and speech confusion. Her superficial lymph nodes, liver, and spleen were not enlarged. Her muscle tone was weak, and muscle strength was diminished. The meningeal stimulation sign was negative. The patient had no notable past medical history. Two months prior, she had experienced dry mouth and occasional gum bleeding, for which she was evaluated at a local hospital. Routine blood work, biochemical tests, and cancer markers were normal, and bone marrow cytology showed no abnormalities. She also had no significant family history or history of heavy alcohol consumption or cigarette smoking.

The laboratory data on admission are as follows. Routine laboratory examinations showed the following: hemoglobin level, 6.4 g/dL [normal range (NR), 11.5–15 g/dL]; total leukocyte count, 2.52×10^9^/L (NR, 4–10×10^9^/L); and platelet count, 26×10^9^/L (NR, 125–350×10^9^/L). Her C-reactive protein level was <6 mg/L (NR, 0–10 mg/L), and erythrocyte sedimentation rate was 156 mm/h (NR, 0–15 mm/h). Further laboratory testing showed high levels of brain natriuretic peptide (BNP; 3,830.70 pg/mL; NR, 0–100 pg/mL), troponin I (0.052 ng/mL), serum lactate dehydrogenase (LDH; 475.09 IU/L; NR, 100–240 IU/L), creatinine (132.70 µmol/L; NR, 45–84 µmol/L), and creatine kinase isoenzyme (56.00 U/L; NR, 0–24 U/L). Her 24-h urinary protein level was 782.99 mg. She tested positive for a direct anti-human globulin test but negative for an indirect anti-human globulin test. A peripheral blood smear showed 0.5% schistocytes. The patient was strongly positive for anti-nuclear antibody, anti-SSA, anti-SSB, anti-RO52, rheumatoid factors, and anti-double-stranded DNA but negative for anti-Smith, anti-cardiolipin, anti-β2 glycoprotein type I, anti-cyclic citrullinated peptide, HLA-B27, anti-myeloperoxidase, anti-protease 3, anti-glomerular basement membrane antibodies, and lupus anticoagulant. Complement C3 and C4 levels were low [0.29 g/L (NR, 0.90–1.80 g/L) and 0.05 g/L (NR, 0.10–0.40 g/L), respectively]. Electrocardiography indicated ST segment changes in some leads, partial lead T wave changes, and a prolonged Q–T interval. Abdominal computed tomography (CT) showed splenomegaly, whereas the size and structure of both kidneys were within normal limits.

SLE was diagnosed according to the American College of Rheumatology/European League Against Rheumatism (ACR/EULAR) classification criteria of 2019 for SLE (3). The SLE disease activity index 2000 (SLEDAI-2K) was 18, indicating severe SLE activity. She was initially treated with a high dose of methylprednisolone (500 mg) for 3 days and gamma globulin (0.4 g/kg/day) for 5 days, followed by immunotherapy with methylprednisolone (60 mg/day). Immunosuppressants such as mycophenolate, cyclophosphamide, calcineurin inhibitors (e.g., cyclosporine, tacrolimus), and azathioprine were viable treatment options for systemic lupus erythematosus involving the hematologic system. Because the patient had significantly reduced total leukocyte count, cyclophosphamide was prone to cause bone marrow suppression, and based on our previous treatment experience, we selected the calcineurin inhibitor tacrolimus(1 mg twice daily). By the 7th day, the patient’s neurological symptoms had completely resolved. Laboratory results showed a total leukocyte count of 6.88×10^9^/L, a hemoglobin level of 9.4 g/dL, a platelet count of 56×10^9^/L, a BNP level of 1691.60 pg/mL, and a troponin I level of 0.027 ng/mL.

On the 30th day, the patient gradually developed dizziness, weakness, and muscle soreness, accompanied by lower limb edema and shortness of breath after activity. CT showed high-density imaging in the upper lobe of the right lung (new), possibly indicating inflammation, bilateral pleural effusion (new), or abdominal effusion. The patient tested negative for the 2019nCoV RdRP gene. The patient was administered ceftriaxone combined with SMZ for infection prevention, methylprednisolone (60 mg) for anti-inflammation, tacrolimus (1 mg, twice daily) for immune suppression, diuretic for cardiac load reduction and blood pressure control, and low-molecular-weight heparin (2,500 U every 12 h) for anticoagulation. Neurological symptoms partially resolved. Re-examination of laboratory indicators showed the following: hemoglobin level, 94 g/L; platelet count, 86×10^9^/L; BNP level, 230 pg/mL; and troponin I level, 0.025 ng/mL. The tacrolimus drug concentration was 1.9 ng/mL. Chest CT showed a decrease in pulmonary infection. On the 35th day, because of abnormal liver function, tacrolimus was replaced with mycophenolate (0.75 g twice daily).

**Figure 1:**
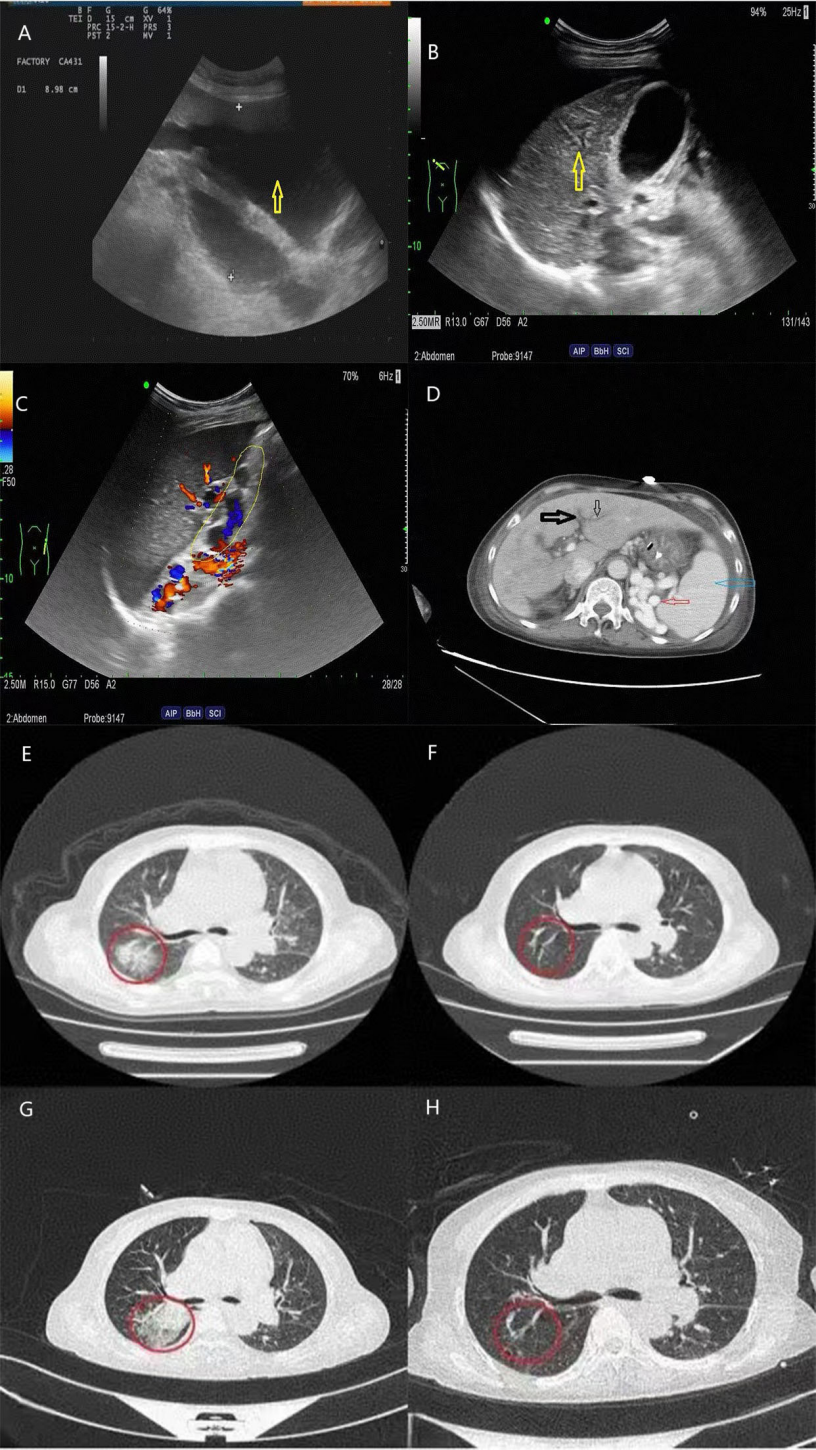
Abdominal ultrasonography and abdominal/chest CT scans of the patient. **(A)** Abdominal color ultrasonography indicates excessive ascites; **(B)** diffuse parenchymal thickening of the liver; **(C)** splenic veins are tortuous and dilated; **(D)** the black arrows show intrahepatic lymphatic dilatation; the blue arrow shows splenomegaly; and the red arrow shows collateral circulation; **(E–H)** show changes in chest CT infection lesions over time.

**Figure 2:**
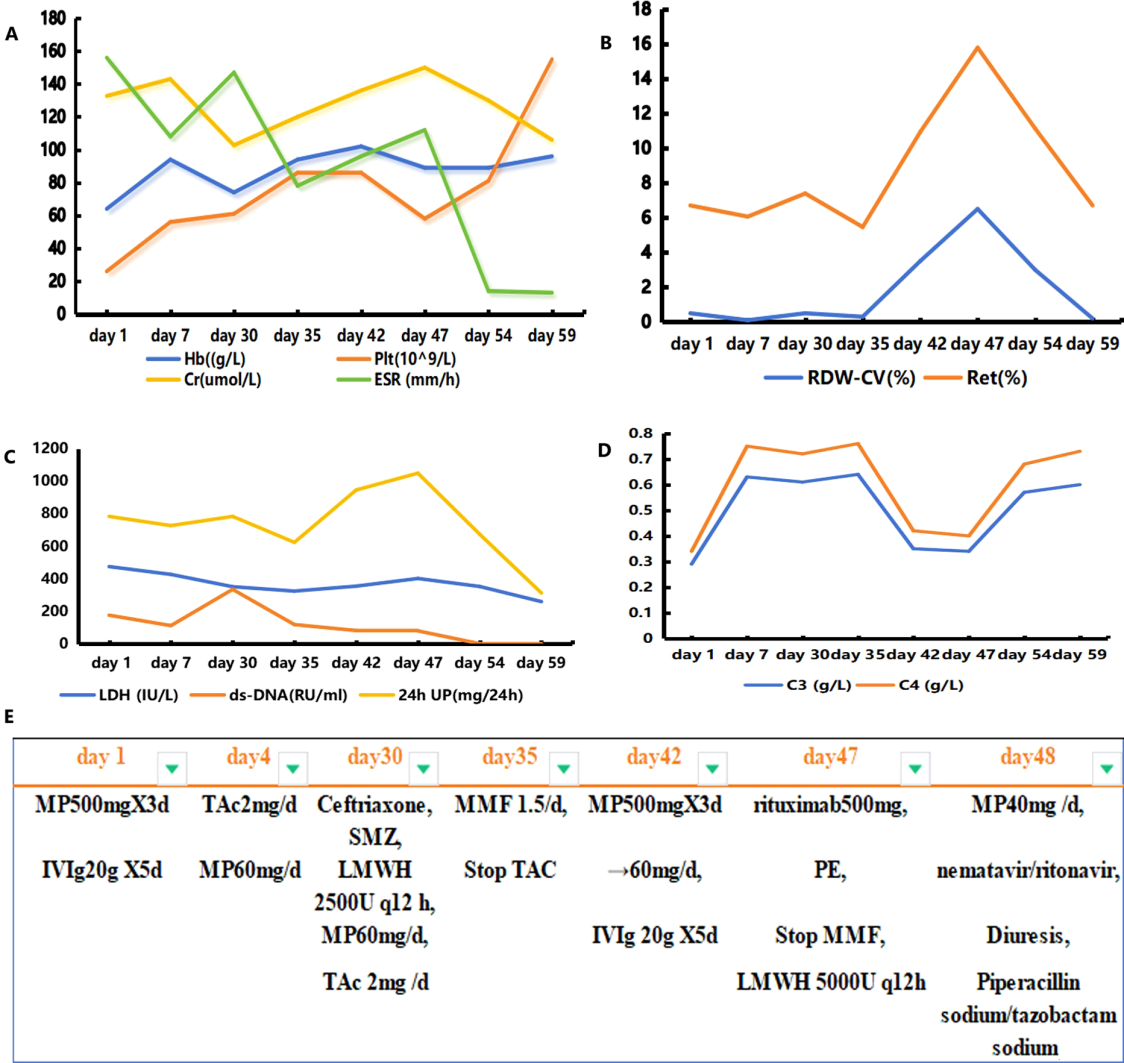
Graph illustrating the evolution of key laboratory parameters and the treatments administered. Panels **(A–D)** represent the changes in the most relevant laboratory parameters over time; panel **(E)** depicts the different treatments administered to the patient. Hb, hemoglobin; Plt, platelet count; RDW-CV, red cell distribution width coefficient of variation; Ret, reticulocyte ratio; Cr, creatinine; ESR, erythrocyte sedimentation rate; 24-h UP, 24-h urinary protein; MP, methylprednisolone; IVIg, gamma globulin; TAc, tacrolimus; MMF, mycophenolate; PE, plasma exchange; LMWH, low-molecular-weight heparin.

On the 42nd day, the patient experienced dizziness and aggravated muscle pain accompanied by low consciousness, slow response, and an inability to understand and complete simple movements. BNP, troponin I, creatinine, and myocardial enzyme levels were all higher than before. The complement level was lower, and the erythrocyte sedimentation rate was higher. She had a SLEDAI score of 18, which indicated severe lupus activity. She was again treated with high-dose methylprednisolone (500 mg) for 3 days and immunoglobulin (0.4 g/kg/day) for 5 days. During the treatment, the patient’s mental symptoms improved, and she was able to understand and complete motor commands. On the 47th day, the patient experienced a recurrence of confusion and delayed responses. Re-examination showed decreased hemoglobin and platelet levels compared with previous values, with peripheral schistocytes at approximately 6.5%. ADAMTS13 activity was 49.18%, ADAMTS13 inhibitor was negative, LDH and creatinine levels increased, and troponin I level increased to 0.087 ng/mL. Abdominal color ultrasonography revealed a large amount of ascites, diffuse liver lesions, splenomegaly, and varicose splenic dilation (**Figure 1**). Enhanced CT revealed diffuse liver disease along the portal veins, intrahepatic lymphatic dilatation, esophageal and gastric varices, a splenorenal vein shunt, and splenomegaly (**Figure 1**). The patient underwent peritoneal puncture and catheterization to drain the turbid yellow ascites. The bacteriological results for the ascites were negative, and cytological pathological examination of the ascites showed no malignant changes. The patient was negative for the spectrum of autoimmune liver disease antibodies, hepatitis virus, tumor markers, and ceruloplasmin. SLE complicated with TMA, and NCPH was considered. She was administered rituximab (500 mg) for immunosuppressive therapy, and mycophenolate was discontinued. Considering the possibility of complement-mediated TMA (CM-HUS) or other atypical HUS, plasma exchange was considered, as it can remove abnormal complement regulatory proteins, CFH antibodies, and other pathogenic factors while simultaneously supplementing normal complement regulatory proteins. The patient underwent plasma exchange (seven times, with 2,000 mL of plasma each time). Low-molecular-weight heparin dosage was increased to 5,000 U every 12 h for anticoagulation. On the 48th day, the 2019nCoV RdRP gene was positive, and nematavir/ritonavir was administered for novel coronavirus treatment. Lung infection lesions increased (**Figure 1**). She was treated with piperacillin sodium/tazobactam sodium for infection, albumin supplementation, diuresis, methylprednisolone (60 mg) for inflammation, and internal environment stability. On the 59th day, the patient’s symptoms and laboratory indices improved (**Figure 2**). Laboratory examinations showed a total leukocyte count of 4.32×10^9^/L, a hemoglobin level of 9.6 g/dL, a platelet count of 155×10^9^/L, a BNP level of 62.40 pg/mL, and a troponin I level of 0.012 ng/mL. C-Reactive protein was <6 mg/L, erythrocyte sedimentation rate decreased to13 mm/h, serum lactate dehydrogenase was 260.46 IU/L, creatinine was 106.00 umol/L, and creatine kinase isoenzyme was 28.00 U/L. The proportion of peripheral schistocytes decreased to 0.2%. She was discharged to the local hospital for continued treatment. At discharge, her medications included prednisone 50 mg/day, aspirin 75 mg/day, pantoprazole 40 mg/day, and calcium carbonate 0.6 g/day, all taken orally. Rituximab (500 mg) was administered again 2 weeks post-discharge after her COVID-19 test turned negative. Six months later, during follow-up, her prednisone dose had been gradually reduced to 10 mg/day, with no recurrence of mental symptoms, normal hemoglobin and platelet levels, and complete ascites resolution.

## 3 Discussion

We identified some clinical issues from the case report. First, infection may be a precursor to SLE disease activity leading to TMA. Chen et al. (4) reviewed the clinical data of 25 patients with SLE complicated with TMA. Among them, 22 patients had moderate-to-severe SLE activity at the time of TMA, suggesting that TMA was mostly parallel to the disease activity of their primary condition. Additionally, 16 patients had concurrent infections, suggesting that infection may be an inducing factor. In patients with SLE, infection can cause TMA either directly or indirectly by inducing lupus activity, with TTP, APS, and complement-mediated TMA being the most relevant causes. However, TMA in lupus patients can also be caused by infection, medications (particularly because of calcineurin inhibitor toxicity), malignant hypertension, or malignancy (5, 6). Calcineurin inhibitors (such as cyclosporine and tacrolimus) can induce dose-dependent endothelial dysfunction, potentially leading to TMA. Although tacrolimus can cause TMA, the patient in this case showed symptoms and laboratory improvement following initial treatment with tacrolimus. On day 35, tacrolimus was discontinued because of abnormal liver function, and mycophenolate was introduced as a replacement. The patient’s condition worsened after discontinuing tacrolimus, but given the low tacrolimus concentration, it is unlikely that tacrolimus induced TMA in this case. In addition, malignant hypertension can cause renal endothelial cell damage, leading to TMA (6). Although the patient had elevated blood pressure at admission, it was stable at the time of clinical worsening, suggesting that malignant hypertension was unlikely to be the primary cause of TMA. Infections are considered the primary cause of lupus activity. In addition to the common Shiga toxin-producing *Escherichia coli*-associated hemolytic uremic syndrome, TMA associated with infections such as those of human immunodeficiency virus, cytomegalovirus, and novel coronavirus has been reported (7). This may be related to direct damage to endothelial cells, complement-mediated damage, or other factors (7). In our case, during the onset of the disease, treatment was effective, but the patient’s symptoms and test indicators worsened after COVID-19 and bacterial lung infections. Her condition did not improve after treatment with high-dose steroids combined with gamma globulin. TMA was considered because of the combination of decreasing hemoglobin and platelet levels, increased LDH and creatinine levels, and a peripheral schistocyte proportion of approximately 6.5%. Infection might have been the inducing factor for the progression of her disease activity to TMA.

Second, patients with TMA and normal ADAMTS13 activity have a poor treatment response. The mechanisms of SLE complicated by TMA are complex and diverse and easily confused with lupus activity, which can impact diagnosis and treatment decisions. Kidney biopsy is the pathological gold standard for TMA diagnosis (8). However, when a kidney biopsy cannot be performed, TMA can be indicated by specific laboratory indicators, such as a decrease in hemoglobin level and platelet count and an increase in LDH level. Before a clinical diagnosis of SLE complicated by TMA, our patient was administered high-dose methylprednisolone combined with immunoglobulin therapy based on her severe lupus activity. However, she showed no improvement in thrombocytopenia, anemia, repeated mental abnormalities, or massive abdominal ascites. Abdominal color ultrasonography revealed disseminated liver lesions, spleen enlargement, and esophageal varicosities. The patient refused to undergo liver or kidney biopsy; however, her microangiopathic hemolytic anemia, thrombocytopenia, elevated LDH level, broken red blood cells on a peripheral blood smear, and portal hypertension indicated SLE combined with TMA. Microthrombus formation can block the hepatic vein or inferior vena cava in the upper segment of the liver, resulting in massive ascites and presenting as Budd–Chiari syndrome (BCS). Anticoagulation is the primary treatment for all cases of BCS in a significantly hypercoagulable state (9). The patient was treated with rituximab (500 mg) and plasma exchange seven times, with 2,000 mL of plasma exchanged each time. The patient’s neuropsychiatric symptoms and laboratory indicators improved. ADAMTS13 activity was normal. ADAMTS13 inhibitor and antiphospholipid antibodies were negative. What type of antibodies could be involved? We suggest that the antibodies may be anti-endothelial antibodies; however, relevant testing was not conducted in this case. We hope that future studies will address similar issues in greater depth and that more scholars will investigate this topic further. The antibodies may be associated with vascular endothelial cell damage induced by SLE, *in vivo* inflammation, and the activation of TMA through complement or other immune pathways. Additionally, SLE-induced dysfunction of the coagulation system may contribute to the occurrence and progression of TMA despite normal ADAMTS13 activity (10). Furthermore, infection could prompt a pro-inflammatory state, activating the clotting and complement cascades, potentially inducing TMA. Patients with TMA secondary to SLE are heterogeneous, and normal ADAMTS13 activity indicates a poor prognosis (11). Plasma exchange can remove cytokines, toxins, and autoantibodies; replace defective plasma factors, and improve TMA symptoms and prognosis in SLE patients. However, the case fatality rate remains high at 27.85%–62.5% (12). Rituximab is an antibody against CD20 that binds to the surface of B cells and eliminates B cells through complement and antibody-dependent cellular cytotoxicity, thereby inhibiting the excessive release of cytokines, reducing endothelial cell damage, and reducing the production of vWF (13). Current guidelines suggest rituximab as a second-line treatment for TMA (14). Furthermore, rituximab can reduce treatment duration and prevent subsequent recurrence (15). From a literature review (2, 11, 16–21), 24 patients with SLE complicated by TMA were treated with rituximab, and most had acute kidney injury as the first symptom; however, in this case, confusion was the initial symptom. Most patients involve only the kidney and nervous system. In this case, the nervous system, blood, kidney, and heart were simultaneously involved, indicating a serious condition and poor prognosis. Rituximab therapy has shown good results, mostly with two or more standard doses combined with high-dose steroids and plasma exchange (**Table 1**). The patient’s peripheral blood B cells were cleared after treatment with 500 mg rituximab, and she demonstrated a positive response to rituximab combined with plasma exchange treatment.

**Table 1:** Summary of patients with TMA secondary to SLE treated with rituximab.

Literature	Number	First presentation	Nerve involvement	Blood system	Renal involvement	SLEDAI	rituximab	Other treatment	Death	Remission	Renal prognosis
Figueiredo CR, et al. (2)	1	Acute kidney injury	0	1	1	NA	375 mg/m^2^×4	High-dose hormone, PE, IVIg, plasma infusion, MMF	0	1	Hematodialysis
Letchumanan, et al. (16)	3	NA	NA	NA	3	17-45	375 mg/m^2^×4(1), 375 mg/m^2^×1(1), 375 mg/m^2^×11(1)	CTX, PE, MMF (3),CsA, VCR (1)	2 (septicemia,empsyxis	2 (1 reappear)	1 recovered
Sun, et al. (12)	13	Acute kidney (7) injury, fever (10)	10	NA	7	15.62 ± 5.88	375 mg/m^2^×4(1), 375 mg/m^2^×2 (12)	High-dose hormone(13), PE (12), IVIg (13)	1	NA	Five hemodialysis
Bu-Hishmeh, et al. (17)	1	Dizziness and flu-like symptoms	1	1	0	NA	375 mg/m^2^×3	High-dose hormone,PE,CTX	0	1	Normal
Limal, et al. (18)	1	1	0	1	1	NA	375 mg/m^2^×4	High-dose hormone,PE	0	1	Normal
Kafle, et al2 (19)	1	NA	1	1	1	NA	2 doses (dosage unknown)	High-dose hormone,PE	0	1	Normal
Kamiya, et al. (20)	1	dyspnea	0	1	1	NA	375 mg/m^2^×4	High-dose hormone,PE	0	1	Normal
Zhou, et al. (11)	1	rash	0	1	1	17	100 mg qw X4次	High-dose hormone, IVIg, dialysis, PE, CTX, MMF, plasma infusion	0	1	Recovered
Xing, et al. (21)	2	Rash (1), cough, sore throat (1)	0	2	2	20	375 mg/m^2^×4(1), 375 mg/m^2^×2(1)	High-dose hormone (2), PE (2), CTX (1), MMF (1)	0	2	Normal(2)

SLEDAI, systemic lupus erythematosus disease activity index; PE, plasmapheresis; IVIg, immunoglobulin intravenous; CTX, cyclophosphamide; MMF, mycophenolate mofetil; CsA, ciclosporin; VCR, vincristine; NA, not applicable; numbers in brackets indicate the number.

Third, portal hypertension can deteriorate concurrently with SLE exacerbation. **Table 2** summarizes the immunosuppressive treatment of NCPH secondary to SLE over the past 25 years (22–28). The age of onset ranged from 19 to 48 years, with most patients being women (six out of seven). Although it took a considerable time for NCPH to manifest as ascites, its progression was rapid once it did. In this instance, NCPH manifested a correlation with SLE activity. The cause of NCPH in SLE remains unidentified; however, several pathophysiological mechanisms have been hypothesized, including immune alterations and hypercoagulability (22). This case suggests that autoimmunity and thrombosis could underlie the development of SLE-associated NCPH. We think that the NCPH and the cerebral and myocardial alterations can be attributed to microangiopathic damage. Hence, some authors suggest steroids as a possible treatment option. In our case, the simultaneous deterioration of SLE hindered glucocorticoid dose reduction. Supplementing anticoagulants with enhanced immunosuppressive therapy may improve the management of portal vein thrombosis, and amelioration of splenomegaly and esophageal varices has been reported after immunosuppressive therapy for SLE. However, every instance occurred in individuals who experienced a brief interval between the onset of SLE and the diagnosis of NCPH (**Table 2**). In our case, at disease onset, the abdominal CT scan revealed an enlarged spleen with no evidence of ascites or any abnormal liver structure. As SLE worsens, NCPH progresses rapidly. It is important to recognize the onset of NCPH early and provide prompt treatment. NCPH treatment includes diuretics, anticoagulants, oral calcium channel blockers, prostacyclin, endoscopic injection sclerotherapy, partial splenic embolization, splenectomy, and devascularization of the stomach vessels (**Table 2**). Most of these treatments improve when SLE is managed concurrently.

**Table 2:** Immunosuppressive treatment of SLE with NCPH reported in the past 25 years.

Literature	Sex/age	Clinical symptoms of NCPH	Clinical symptoms of SLE	Interval between SLE and INCPH	Hepatichistopathology	Treatment SLE	Treatment NCPH	Outcome of NCPH	Outcome of SLE
Keisuke Imabayashi (22)	F/43	A large amount of ascites, splenomegaly, a hypoechoic band in the liver	Pancytopenia, retinalvasculitis, panniculitis, cholecystitis, and enteritis	Simultaneously	No features of microthrombus, liver cirrhosis, parasite infection or regenerative nodules	PSL 50 mg, IVCY	Diuretics, anticoagulant agent	Ascites improved, Unchanged (splenomegaly, esophageal varices, the hypoechoic band)	Improved
Park YW et al. (23)	F/37	Splenomegaly, esophageal varices	Pancytopenia	Simultaneously	NRH	PSL (1.5 mg/kg)	Oral calcium channel blocker and prostacyclin	Unchanged	Improved
Silvia Suárez-Díaz et al. (24)	F/19	Splenomegaly, esophageal varices	Discoid erythema、 arthritis、 proteinuria	11 years	No portal or sinusoidal fibrosis	PSL, HCG, rituximab 2 g, CTX	No special treatment	Died	Died
Yamamoto M et al. (25)	F/48	Splenomegaly	Pancytopenia, polyarthritis, nephritis	Simultaneously	NA	PSL 40 mg pulse HCQ	No special treatment	Improvement (splenomegaly and enlarged portal vain)	Improved
Yang QB et al. (26)	F/48	Splenomegaly, esophageal varices	Pancytopenia, polyarthritis, hepatitis	Simultaneously	Autoimmune hepatitis	PSL 40mg	No special treatment	Improvement (splenomegaly and esophagealvarices)	Improved
Horita T et al. (27)	F/40	Esophageal varices	Nephritis, serositis, fever	16 years	NRH	PSL	Endoscopic injection sclerotherapy	Died (bacterial endocarditis)	Died
Inagaki H et al. (28)	M/20	Splenomegaly, esophageal varices	Nephritis	18 years	Periportal fibrosis and collapse of peripheral portal vein branches	Splenectomy (no response to mPSL pulse therapy)	partial splenic embolization, splenectomy, and devascularization of the stomach vessels	Improvement (esophageal varices)	Improved

PSL, prednisolone; NRH, nodular regenerative hyperplasia; NA, not available; HCQ, hydroxychloroquine; IVCY, intravenous cyclophosphamide pulse therapy.

This case suggests that rituximab, combined with plasma exchange, anticoagulation, and diuretics, may be an effective therapeutic option for patients with SLE complicated by TMA and NCPH. However, the pathogenesis and prognosis of SLE in the context of TMA and NCPH remain complex.

## Data Availability

The original contributions presented in the study are included in the article/supplementary material. Further inquiries can be directed to the corresponding authors.
